# Genetic characterization of *Cryptosporidium* spp. among patients with gastrointestinal complaints 

**Published:** 2016

**Authors:** Reza Ranjbar, Kaveh Baghaei, Ehsan Nazemalhosseini Mojarad

**Affiliations:** 1 *Molecular Biology Research Center, Baqiyatallah University of Medical Sciences, Tehran, Iran.*; 2 *Gastroenterology and Liver Diseases Research Center, Research Institute for Gastroenterology and Liver Diseases, Shahid Beheshti University of Medical Sciences, Tehran, IR Iran *

**Keywords:** Genetic characterization, *Cryptosporidium*, Gastrointestinal complaints

## Abstract

**Aim:**

This study investigated subtypes of *Cryptosporidium* in patients with gastrointestinal complaints in Tehran, Iran.

**Background:**

*Cryptosporidium*, an intracellular protozean parasite, is among the major causative agents of gastroenteritis disorders in humans. It also causes water-borne and food-borne outbreaks of diarrheal diseases.

**Patients and methods:**

A total of 1685 fecal samples were collected from patients with gastrointestinal complaints who had been referred to clinical laboratories Tehran, Iran. The primary diagnosis was established by the detection of oocysts using the modified Ziehl-Neelsen staining method and following that, the positive microscopically samples were selected for sequence analysis of the partial 60 kDa glycoprotein (gp60) gene.

**Results:**

Out of 1685 collected samples, 7 (0.4 %) were positive for *Cryptosporidium* oocysts. Sequence analysis of gp60 gene in seven *Cryptosporidium* isolates revealed that two subtype families were identified, IIa and IId. Five (of 7) isolates belonged to the subtype family IIa and the remaining two isolates belonged to IId. Two sub-types were recognized within the subtype family II,a including IIaA16G2R1 (3/5), IIaA17G1R1 (2/5), while IIdA17G1d was the only subtype within IId subtype family.

**Conclusion:**

The predominance of zoonotic subtype families of *C. parvum* species (IIa, IId) in this study highlights the importance of *zoonotic* transmission of cryptosporidiosis in the country.

## Introduction

 Intestinal protozoan parasites are still major health problems in tropical and subtropical areas and are characteristically found among people with a low socio-economic grade and poor hygiene (1). Among these intestinal parasites,* Cryptosporidium *spp. is a main pathogen which is a cause of diarrhea in children and immunocompromised patients and also infects the gastrointestinal tract of a wide range of vertebrates, including domestic and wild animals and birds (2-4). 

The parasite is transmitted through the faecal-oral route, following direct or indirect contact with *Cryptosporidium* oocysts via person-to-person, zoonotic, contaminated water, foodborne or airborne contact (5-7). 

Currently, the use of molecular approaches in genetic characterization of *Cryptosporidium* spp. at polymorphic loci has allowed a better understanding of the epidemiology of cryptosporidiosis (2-3). Molecular biology has well-known as a powerful tool for categorizing *Cryptosporidium *and has discovered major variation within the genus (about 30 species). Two predominantly species that have been found in humans are *C. parvum *and *C. hominis*. However, other species such as *C. meleagridis*, *C. muris*, *C. felis*, *C. canis*, *C. suis *and *C. andersoni *have been rarely detected in feces of immunocompetent and immunocompromised individuals (8).

In the past two decades, reports of cryptosporidiosis in Iran have been achieved using microscopy (9-13). Recently, researchers have developed PCR-based techniques for detection and identification of *Cryptosporidium *spp. (14-17).

Fingerprint of *C. parvum* infection has a critical role in outbreak investigations. DNA sequencing of the *Cryptosporidium* 60-kDa glycoprotein (GP60) gene has revealed substantial genetic heterogeneity among *C. hominis* and *C*. *parvum* isolates. GP60 gene could be used as a marker to determine the different subtype families within both species, including: Ia, Ib, Id, Ie, If and Ig for *C. hominis* and IIa, IIb, IIc, IId, IIe, IIf , IIg, IIh, IIi, IIk, and IIl for *C.* *parvum* (18). Within each subtype group, there are several subtypes primarily based on the number of tri-nucleotide repeats coding for the amino acid serine (2, 7, 19).

To our knowledge, there are several molecular epidemiological studies that have documented the distribution of subtypes of *cryptosporidium* ssp. in children with diarrhea (8, 15), animals (20) and environments (the water that isolated from rivers) (21) in Iran. 

In this study, we identified the genotypes of the *Cryptosporidium* isolates from patients with gastrointestinal complaints referred to clinical laboratories of Tehran using the polymerase chain reaction (PCR) amplification and sequencing analyses of the *Gp 60 *gene. 

## Patients and Methods

**Sampling**

A total of 1685 fecal samples were collected from patients with gastrointestinal complaints who had been referred to Medical Centers in Tehran, Iran. *Cryptosporidium* oocysts were identified in samples after concentration by formalin–ethyl–acetate sedimentation and staining with a modified Zeihl-Neelsen technique (22). The positive *Cryptosporidium* spp. isolates were preserved in 2.5% potassium dichromate and kept at 4°C until DNA extraction.

**DNA extraction**

Approximately 300 μl of fecal suspension was washed three times with distilled water to remove trace of dichoromate and then genomic DNA was extracted using DNAzol kit according to the manufacturer’s instructions (Invitrogen, life technologies, Cat. No 10503-027, USA) with the addition of three freeze-thaw cycles (10 minutes) after resuspending samples in lysis buffer (to rupture the *Cryptosporidium* oocysts). The oocysts were frozen in the liquid nitrogen tank. Thawing was carried out at 90° C in the water bath.

**DNA subtyping and sequence analysis**

For subtyping *C. parvum* and *C. hominis*, a fragment of about 400 bp of the gp60 gene was amplified by nested PCR with the primers 5_-ATA GTC TCC GCT GTA TTC-3_ and 5_-GCA GAG GAA CCAGCA TC-3_ in primary PCR and 5_-TCC GCT GTA TTC TCA GCC-3_ and 5_-GAG ATA TAT CTT GGT GCG-3_ in secondary PCR, as described (Abe et al., 2006). PCR products were visualized by electrophoresis in 1.5% agarose gels stained with ethidium bromide.

The PCR-amplified products were subjected to direct sequencing using a BigDye Terminator Cycle Sequencing Kit (Applied Biosystems, Foster City, CA) and a Genetic Analyzer PrismTM 3130x1 (Applied Biosystems). The secondary PCR products were sequenced in both directions and, if variations were found, results were confirmed by sequencing of at least two independent PCR products. All sequences were edited manually and analyzed with reference sequences using the GenRunner software (v. 3.05).

Subtypes were recognized based on the number of trinucleotide repeats (TCA or TCG) coding for the amino acid serine (19).

**Statistical analysis**

 The prevalence of *Cryptosporidium* infection and prevalence of *C*.* parvum* and *C. andersoni* in pre-weaned calves was compared with prevalence data for post-weaned calves. The Chi-square test for independence was used to analyze the data and differences were considered significant when P < 0.05. Statistical analysis was performed using SPSS (Ver. 12). 

## Results

**Figure 1:**
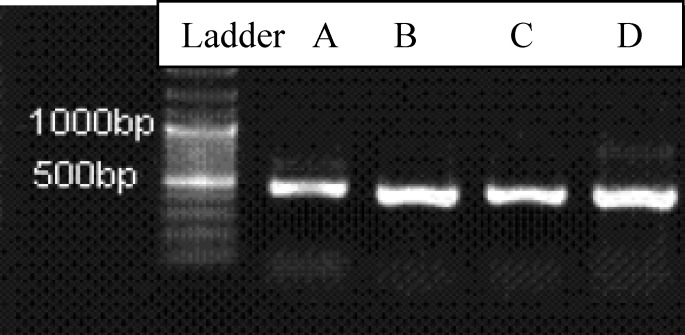
PCR of *Cryptosporidium *based on *GP60 *gene. Lane 1: 100 bp DNA marker, lane A-D: *Cryptosporidium ssp.*

Among the 1685 patients (66.3% male and 33.7% female) included in this study, microscopic examinations of the specimens revealed the presence of *Cryptosporidium* oocysts in 7 (0.4 %) of the samples. Clinical information about these samples is presented in table 1. Patient complaints included abdominal pain, flatulence, tenesmus, diarrhea and dysentery. All samples were successfully amplified using specific primers (Figure 1) and PCR products of GP60 gene were purified and sequenced using a genetic analyzer machine.

The sequences were determined and analyzed using the chromas program and aligned with each other and with previously reported sequences for identification of the alleles and subtypes. The result of this analysis showed that all isolates were *C*.* parvum* species. 

**Table 1:** Distribution of *Cryptosporidium parvum *subtypes in isolates from patients with gastrointestinal complaints

ID	Subtype Family	Subtype	Sex/Age	GI complaints	Location
1	IIa	IIaA16G2R1	M\ 49	abdominal pain, tenesmus	Tehran
2	IIa	IIaA16G2R1	F\13	dysentery , abdominal pain	Varamin
3	IIa	IIaA16G2R1	M\ 32	abdominal pain	Pakdasht
4	IIa	IIaA17G1R1	M\27	dysentery , abdominal pain, flatulence	Tehran
5	IIa	IIaA17G1R1	F\ 52	abdominal pain	Pakdasht
6	IId	IIdA17G1d	M\11	dysentery , abdominal pain	Varamin
7	IId	IIdA17G1d	F\68	dysentery , abdominal pain, flatulence	Pakdasht

The sequence analysis of gp60 gene in seven *Cryptosporidium* isolates revealed that, two subtype families were identified, IIa and IId. Five (of 7) isolates belonged to the subtype family IIa and remaining two isolates belonged to IId. Two sub-types were recognized within the subtype family IIa including IIaA16G2R1 (3/5), and IIaA17G1R1 (2/5), while IIdA17G1d was the only subtype within the IId subtype family. 

## Discussion

There are wide intraspecific variations in *C*.* parvum* populations. Currently, at least 14 *C*.* parvum* subtype families, including IIa, IIb, IIc, IId, IIe, IIf, IIg, IIh, IIi, IIk, IIl, IIm, IIn, and IIo, have been identified on the basis of sequence analysis of the 60 KDa glykoprotein (GP60) gene (23).

In this study, *Cryptosporidium* isolated were classified by sequence *analysis* of the 60-kDa glycoprotein (*GP60*) *gene*. A prevalence rate of 0.4 % (7/1685) was obtained for cryptosporidiosis among patients with gastrointestinal complaints.

All *Cryptosporidium* isolates from patients with gastrointestinal complaints were identified as *C. parvum* species and none of them belonged to *C. hominis*. Also, two main subtype families (IIa and IId) were recognized. Regarding to other studies, *C. parvum* is reported in most cryptosporidiosis cases in Iran (6,14,24), which highlights the significance of zoonotic transmission of cryptosporidiosis in the country (25-26).

Although, in some countries such as Australia, India, Egypt, Mexico and Peru, predominance of *C. hominis* in human isolates have been documented (27-31), but our result is consistent with studies from some other countries such as Malaysia, Kuwait, Yemen, Sweden, United Kingdom, Netherland, France, Portugal, and Nicaragua ( 32-39)

First characterization of *Cryptosporidium* subtypes in Iranian specimens was documented in 2011. According to their results, 47 samples of *C*.* parvum* and *C. hominis* were characterized in children and cattle by sequence analysis of the gp60 gene, which showed cattle and children were mainly infected by *C. parvum* IIa subtypes and *C.* *parvum* IIa and IId subtypes, respectively (17). In some countries such as Spain, Egypt and China, IId subtypes are known to be more prevalent in sheep and goats (40-42). Also, IId subtypes are also common in children from Iran neighboring countries (43-46). Sharbatkhori and her collogues, showed three haplotypes of IIa subtype family including IIaA16G2R1, IIaA17G1R1, IIaA22G3R1 and one haplotype of IId subtype family among diarrheic children from Gonbad Kavoos City (Golestan Province, Northern Iran) and suggested a zoonotic transmission of cryptosporidiosis in this area (8). 

The majority of IIa and IId subtypes highlight the significance of zoonotic *Cryptosporidium* transmission in Iran. Thus, cattle could be a plausible source of human infection with *C*.* parvum* IIa in Iran (15-18). 

In another study, in 2011, high diversity of *Cryptosporidium* sub-genotypes was shown among Malaysian HIV infected individuals. The results obtained from this paper signified the possibility of zoonotic as well as anthroponotic transmissions of cryptosporidiosis in HIV infected individuals (47).  In 2015, Mahmoudi, et al.   determined the genotype and subtype distribution of *Cryptosporidium* spp. in river water samples in Iran. They showed that all *C. parvum* and *C.* *hominis* isolates belonged to the IId and Id subtype families, respectively and this source is a potential risk of waterborne cryptosporidiosis in humans and animals (21).

Vieira, et al. identified two subtype families (IIa and IId) from children, calves and eight pigs in Romania. They proposed, cattle might be the source of *Cryptosporidium* infections for humans and the transmission dynamics of *C. parvum* in Romania (48).

In their study, Wang, et al. suggest that, due to the higher nucleotide diversity of *C*.* parvum* IId GP60 sequences, more population genetic studies using high-resolution tools are needed to present a better explanation of the origin and dissemination of *C. parvum* in the world (23).

In 2010, sequence analysis of the GP60 locus identified three *C. parvum* and two *C. hominis* subtype families in Jordan. In this study several rare and novel subtypes were reported as well (45).

Preliminary molecular epidemiological studies have revealed some unique features of cryptosporidiosis transmission in humans in Iran and other Mideast countries. As the *C. parvum* subtype family IId was the dominant family causing cryptosporidiosis in humans in Iran, zoonotic transmission could possibly be involved. However, more extensive sampling of both humans and farm animals, especially sheep and goats, and collection of epidemiological data in case-control and longitudinal studies are needed for a better understanding of the sources of *C. parvum* infections in humans in Iran and other Mideast countries.
